# Association Between Endothelial Cell Stabilizing Medication and Small Vessel Disease Stroke: A Case-Control Study

**DOI:** 10.3389/fneur.2019.01029

**Published:** 2019-09-25

**Authors:** Charlotte Elisabeth Becker, Terence J. Quinn, Anna Williams

**Affiliations:** ^1^Centre for Regenerative Medicine, UK Dementia Research Institute, University of Edinburgh, Edinburgh, United Kingdom; ^2^School of Medicine and Health Sciences, University of Oldenburg, Oldenburg, Germany; ^3^Institute of Cardiovascular and Medical Sciences, University of Glasgow, Glasgow, United Kingdom

**Keywords:** small vessel disease, endothelial cell, stroke, statin, lacunar, anti-hypertensive

## Abstract

Increasing evidence suggests a role for endothelial cell (EC) dysfunction in pathogenesis of cerebral small vessel disease. Commonly used medications including certain antihypertensives and statins have EC-stabilizing effects. We used individual patient data from completed acute stroke trials to assess whether prior exposure to EC-stabilizing medications was associated with lacunar stroke, using lacunar stroke as a clinical proxy for cerebral small vessel disease. Across 12,002 patients with relevant data, 2,855 (24%) had a lacunar stroke presentation. Univariable analyses suggested potential confounding from vascular diseases treated with EC-stabilizing medications. Initial multivariable logistic regression gave conflicting results when describing the independent association of exposure to EC-stabilizing medication and lacunar stroke in the complete population (O.R. 0.87, 95% C.I.: 0.77– 0.98) and limited to those taking any antihypertensive (O.R. 1.51, 95% C.I.: 1.21–1.88). Re-running the analyses including statins in the EC-stabilizing category suggested a beneficial effect of EC-stabilizing medication exposure on lacunar stroke incidence (O.R. 0.83, 95% C.I.: 0.73–0.93). These results align with recent pre-clinical data and would support interventional trials of EC-stabilizing medication for preventing cerebral small vessel disease. Our results also suggest that analyses of EC-stabilizing interventions need to adjust for potential endothelial effects of other co-prescribed medication.

## Introduction

Cerebrovascular small vessel disease (SVD) is common, increases with age (1) and accounts for almost half of all dementias (2), one fifth of all strokes (3), especially lacunar strokes, and more than four fifths of all intracerebral hemorrhages (4). Neuropathological features of SVD include progressive changes in the perforating arterioles, capillaries, and venules, eventually causing cerebral white and deep gray matter damage (5, 6), and accompanied by characteristic neuroradiological changes on brain imaging (7, 8).

Recently, mechanistic links have been shown between dysfunction of endothelial cells (EC) in cerebral small blood vessels and white matter damage in a rat model of SVD and also in human SVD tissue (9). EC dysfunction is defined as a state where there is less available NO, more proliferation, reduced tight junctions between cells and increased production of HSP90a (10). SVD patients have elevated plasma levels of biomarkers of EC dysfunction, such as ICAM-1 (11) and a reduced vasodilatory response to vasoactive challenges (12). The pathological changes of EC dysfunction and white matter damage in a rat model of SVD were reversed by use of drugs known to stabilize endothelial function, by increasing nitric oxide production (9). EC-stabilizing drugs included perindopril (an Angiotensin Converting Enzyme Inhibitor) and simvastatin (a HMG Co-A reductase inhibitor), both are drugs that are in common use in clinical practice.

One question raised by this work is whether SVD is also reversible in humans, and clinical trials are in progress which may help answer this (LACI-1 (13, 14) and LACI-2) (Clinical trials: ISRCTN12580546, ISRCTN14911850). These trials have chosen drugs reversing endothelial dysfunction, due to the hypothesis that SVD is not primarily caused by hypertension (15) or atheroma (16). Classes of drugs with potential endothelial benefit have been used to reduce cardiovascular risk for many years, and so this provides an opportunity to examine clinical data as a first step to answering this question. Trials of antihypertensives in reducing SVD-related strokes have shown conflicting results, with some showing a benefit and others not [reviewed in (17)]. This may be in part as the hypothesis tested was whether lowering blood pressure (using any medication) reduces incidence of lacunar stroke. Within the antihypertensive rubric are various drug classes with differing modes of action and not all will have endothelial effects. An alternative question is whether medications that stabilize endothelial cells or reverse their dysfunction regardless of their effect on hypertension are effective in reducing SVD. In support of this, trials using angiotensin converting enzyme inhibitors (18) or statins (19) (both EC-stabilizing) have shown reduction in the progression of neuroimaging features of SVD. The overall contribution of classical vascular risk factors to SVD burden is modest and we feel that there is a need for research looking at novel risk factors (20) and that recognizes the interaction of vascular disease with other processes (21).

We utilized existing stroke trial data to test our hypothesis that use of medications with EC-stabilizing function is associated with reduced SVD. As SVD is often clinically covert, we have used the more clinically obvious manifestation of cerebral SVD of lacunar stroke. We firstly compared incidence of lacunar stroke between patients exposed to antihypertensives with and without EC-stabilizing properties. We then assessed association of lacunar stroke with exposure to statins, which also are known to stabilize EC function without altering blood pressure.

## Materials and Methods

We created a case-control experiment with two groups: patients taking medications associated with EC-stabilizing effects (defined using various criteria as described below) and those not taking these drugs. We analyzed the association of prior exposure of EC-stabilizing drugs using clinical lacunar strokes as a proxy for SVD. Using lacunar stroke events as a proxy for SVD is attractive as it can be determined from stroke records in retrospect, with typical clinical features of pure motor hemiparesis, pure sensory syndrome, sensorimotor stroke, ataxic hemiparesis, or dysarthria-clumsy hand.

### Dataset

We used the Virtual International Stroke Trials Archive (VISTA) as our data source. VISTA is a not-for-profit organization that archives anonymized, patient level data from completed stroke trials (22). We extracted individual patient data from the VISTA resource that allowed characterization of stroke type, pre-stroke medication history, cardiovascular risk factors, age, sex, and mortality. The data were taken from completed acute stroke trials and so all included patient data were from patients with a stroke event. The use of fully anonymized data from VISTA for novel research purposes has Institutional ethical approval (University of Glasgow, MVLS ethics). These data were all from studies within this involving human participants that were in accordance with the ethical standards of the institutional and national research committee and with the 1964 Helsinki declaration and its later amendments, including informed consent.

### Exposure

The first exposure of interest was antihypertensive therapy prior to stroke. We defined drugs that stabilize EC function as those known to increase the production of nitric oxide (NO) by finding any peer-reviewed publication with evidence of this by examination of the literature (Supplementary Table 1). Drugs were defined separately, rather than in their groups, as for example, some betablockers are known to be EC-stabilizing and others are not. Each of the approximately 168,000 medications contained in our VISTA file was classified, by evidence in the literature, into:

1. Antihypertensive medications that stabilize ECs
2. Antihypertensive medications that do not stabilize ECs
3. Mixed preparations (drug combinations with stabilizing and non-stabilizing effects)
4. Non-antihypertensive medications without EC-stabilizing effects

Group 1 and 3 medications were grouped together as EC-stabilizing. Where a patient was taking more than one antihypertensive drug, if any belonged to the EC-stabilizing group, then the patient was classified into the EC-stabilizing antihypertensive exposed group. Patients taking these agents for reasons other than hypertension, such as migraine, arrhythmia, or benign prostate hyperplasia were included as we were interested in endothelial effect rather than blood pressure effect *per se*. Only oral agents were considered. As a secondary analysis, we included statins as medications that stabilize EC function, though without anti-hypertensive properties. Exposure to any statin was classified as EC-stabilizing. The dose and duration of these medications for each patient was not available. Thus, any exposure was included.

A list of the statins, antihypertensive drugs and their categorization is provided in Supplementary Tables 1, 2.

### Outcome

**Figure 1:**
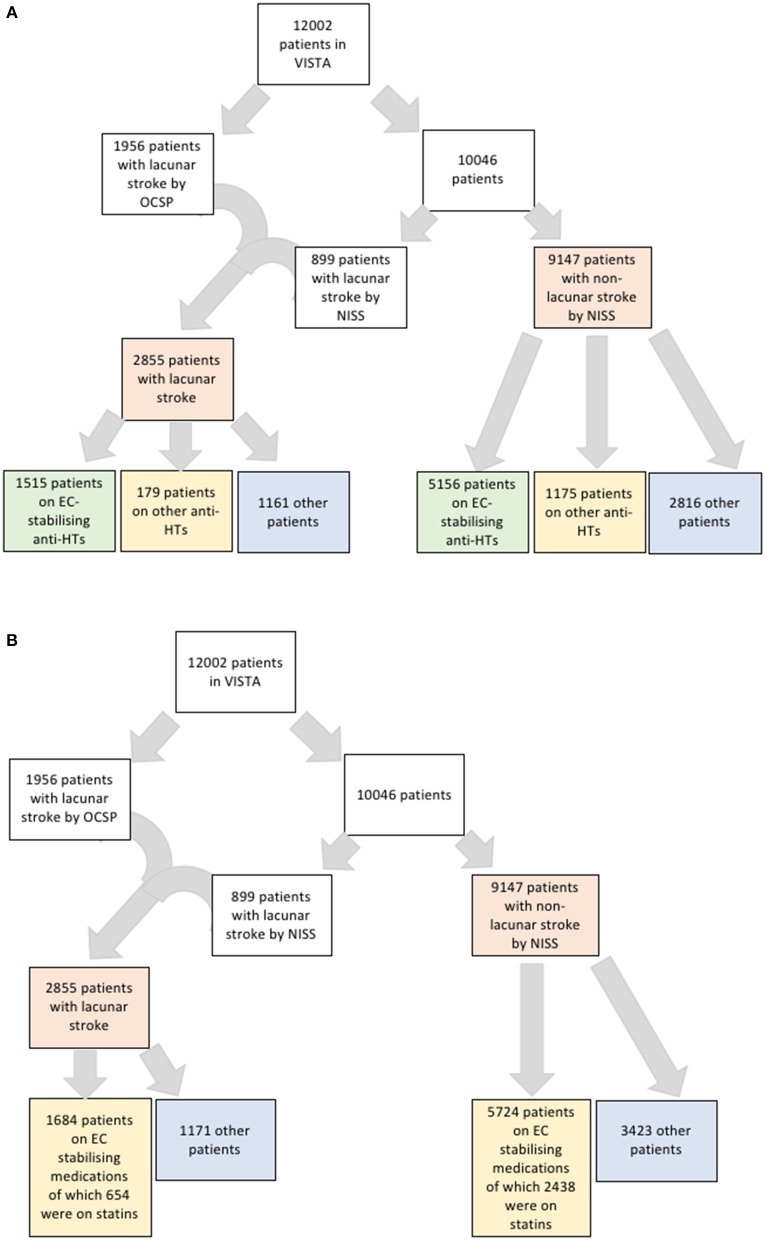
**(A)** Flowchart of strategy for identifying patients with lacunar stroke and their numbers on anti-hypertensives that are endothelial cell-stabilizing or not. **(B)** Flowchart of strategy for identifying patients with lacunar stroke and their numbers on endothelial cell-stabilizing medications including statins.

The outcome of interest was lacunar stroke, the most recognizable clinical manifestation of SVD and used here as a proxy for SVD. We used a multimodal approach to classify patients as having lacunar or non-lacunar stroke. We used the Oxfordshire Clinical Stroke Project classification (OSCP) (23) to categorize stroke type retrospectively and in particular to identify lacunar strokes (LACS). Where the OCSP classification was absent, we used the National Institute of Health Stroke Scale (NIHSS) to exclude patients on the basis of cortical impairments that were not compatible with a lacunar stroke presentation. We ensured that the final selection of patients all had a clinical presentation in keeping with a recognized lacunar stroke syndrome: pure motor, pure sensory, or pure sensory-motor (Figure 1). Radiological data were not used.

### Analyses

In our initial analyses, we classified EC-stabilizing medication exposure based on antihypertensive medication only. Our analyses were based on a protocol that was reviewed and approved by the VISTA steering committee. We made the a-priori decision to use multivariable logistic regression for our primary analyses as we felt that this approach made the fewest assumptions and allowed greatest use of the available data. We performed initial univariable proportional (chi square) analyses to describe associations between exposure to EC-stabilizing medication and lacunar stroke. We then used multivariable models to correct for potential clinical and demographic confounders, specifically focusing on those conditions associated with prescription of a particular antihypertensive. Co-variables of interest were: age; sex; history of hypertension; atrial fibrillation; myocardial infarction; diabetes mellitus; and previous stroke. Choice of variable for inclusion in the multivariable model was based on biological plausibility and previous research on small vessel disease. As per best practice in these analyses, the choice for inclusion was not based solely on univariate associations. However, there were no data available for how well co-morbid conditions were controlled. We employed backwards logistic regression against an outcome of lacunar stroke and presented the resulting data as odds ratios (O.R.) and associated 95% confidence intervals (C.I.). As a further adjustment for the potential indication bias of antihypertensive medication, we re-ran the univariable and multivariable analyses limiting the included population to those taking antihypertensive medication.

We repeated the multivariable analyses to study the effect of statin exposure. Firstly, we added statin exposure into the multivariable model. We then re-ran the multivariable analysis, creating a new exposed population that included both patients taking EC-stabilizing antihypertensives and statins. Finally, we performed *post-hoc* exploratory analyses to study differences in 90 day mortality between exposed and non-exposed groups, where the exposed group was firstly those taking EC-stabilizing antihypertensives and then those taking EC-stabilizing antihypertensives and/or statins. For these binary regression models, we calculated that we needed at least 90 outcomes (lacunar stroke) for a model that contained all nine variables of interest and thus we were confident that our sample had adequate power for primary and all subgroup analyses. All analyses were performed using *SPSS (Version 1.0.0.7, IBM)*.

## Results

Our dataset included 12,002 patients taking 168,000 medications. Of these patients, we defined 2,855 (24%) as having lacunar strokes. Of these patients, 2,276 had NIHSS data suitable for analysis for the subtype of lacunar stroke with 1,020 sensorimotor, two pure sensory, 735 pure motor, 205 ataxic hemiparesis, one dysarthria-clumsy hand, and 313 other. The small number of pure sensory strokes likely relates to RCT inclusion criteria, where a minimum level of NIHSS would be needed to be eligible for inclusion. However, we did not subdivide the lacunar strokes types in further analysis. Of the total included patients, 8,025 patients had been exposed to antihypertensive medications (6,671 to antihypertensives that stabilize EC function and 1,354 as exposed to antihypertensives that do not stabilize EC function) and 3,978 were not taking any antihypertensive medication (Figure 1A). Of the 9,147 patients with non-lacunar strokes, there was information for stroke subtype for 9,089 patients, and 8,652 patients had an ischaemic stroke and 437 a haemorrhagic stroke.

First, we asked whether, in the entire dataset, there was a difference in the proportion of lacunar strokes between patients taking EC-stabilizing antihypertensive medications prior to stroke and those who are not. On unadjusted testing including all patients, there was a lower proportion of lacunar stroke in those exposed to EC-stabilizing antihypertensive medication (Table 1). This was in spite of the higher incidence of diabetes mellitus, atrial fibrillation, previous stroke and hypertension in the patients taking EC-stabilizing antihypertensives. However, when we confined the analysis to the population treated with any antihypertensive medications, there were more lacunar strokes found in patients taking EC-stabilizing antihypertensives (Supplementary Table 3). Again, this treatment group was more likely to have been diagnosed as having hypertension and diabetes mellitus.

**Table 1:** Univariable analysis comparing all patients exposed or not to endothelial cell-stabilizing antihypertensive medication.

	**Exposed to EC-stabilizing anti-HT drug *N* = 6,671**	**Not-exposed to EC-stabilizing anti-HT drug *N* = 5,331**	***P***
Female	3,024	2,314	0.03
Hypertension history	4,840	2,342	<0.0001
Diabetes Mellitus	1,456	796	<0.0001
Myocardial Infarct	833	584	0.23
Atrial fibrillation	1,649	981	<0.0001
Previous stroke	1,400	1,413	<0.0001
Lacunar stroke	1,515 (23%)	1,340 (25%)	0.002
Non-lacunar stroke	5,156 (77%)	3,991 (75%)	

*EC, Endothelial cell; HT, hypertensive*.

*The final cell reports significance testing for comparative analysis comparing proportion of lacunar stroke in those exposed and non-exposed to EC stabilizing antihypertensive medication*.

Due to the possible confounding of vascular conditions that may be treated with EC stabilizing medication, we used multivariable logistic regression to determine whether exposure to EC stabilizing antihypertensives was independently associated with lacunar strokes in both the entire dataset and in the group taking any antihypertensive medication as before. Comparing those on an EC-stabilizing antihypertensive with the rest of the dataset, the O.R. of having a lacunar stroke was significantly less if the patient was taking an EC-stabilizing antihypertensive (O.R. 0.87, 95% C.I.: 0.77–0.98; Table 2). When repeating the analysis confined to only those patients taking antihypertensive medications the O.R. of having a lacunar stroke was significantly greater if the patient was taking an EC-stabilizing anti-hypertensive (O.R. 1.51, 95% C.I.: 1.21–1.88; Table 3).

**Table 2:** Multivariable model (logistic regression) describing odds ratios of lacunar stroke including all patients.

	**Odds ratio (95% CI)**	***P***
Increasing age (+1 year)	0.98 (0.98–0.98)	<0.0001
Male	1.07 (0.96–1.20)	0.25
Hypertension history	1.03 (0.90–1.18)	0.63
Diabetes Mellitus	1.05 (0.92–1.20)	0.51
Myocardial Infarct	0.81 (0.69–0.96)	0.015
Atrial fibrillation	0.48 (0.41–0.56)	<0.0001
Previous stroke	1.82 (1.6–2.1)	<0.0001
EC stabilizing anti-HT drug	0.87 (0.77–0.98)	0.017

*EC, Endothelial cell; HT, hypertensive*.

**Table 3:** Multivariable model (logistic regression) describing odds ratios of lacunar stroke including only patients exposed to antihypertensive medication.

	**Odds ratio (95% CI)**	***P***
Increasing age (+1 year)	0.97 (0.97–0.98)	<0.0001
Male	1.01 (0.87–1.18)	0.86
Hypertension history	1.24 (1.02–1.5)	0.03
Diabetes mellitus	0.95 (0.8–1.13)	0.58
Myocardial Infarct	0.87 (0.7–1.08)	0.22
Atrial fibrillation	0.49 (0.4–0.59)	<0.0001
Previous stroke	1.75 (1.5–2.0)	<0.0001
EC stabilizing anti-HT drug	1.51 (1.21–1.88)	<0.0001

*EC, Endothelial cell; HT, hypertensive*.

To assess these seemingly inconsistent results, we considered the possibility that statins, which have EC-stabilizing properties but no antihypertensive effect, may be a confounding factor. In our dataset, a total 3,092 patients were taking statins (Figure 1B). Firstly, we added statin exposure as a variable to the multivariable model using the complete dataset. In this revised analysis, statins were independently associated with a reduction in the likelihood of lacunar stroke (O.R. 0.73, 95% C.I.: 0.63–0.84) while the association between EC stabilizing antihypertensive medication and reduction in lacunar stroke was lost (Table 4). When we re-ran the multivariable model with a new exposure classification that included both patients exposed to relevant antihypertensive medication and statins, then the EC-stabilizing medication exposed group had a significantly lower O.R. of having a lacunar stroke (O.R. 0.83, 95% C.I.: 0.73–0.93; Table 5).

**Table 4:** Multivariable model (logistic regression) describing odds ratios of lacunar stroke including all patients and adding statin exposure as a variable.

	**Odds ratio (95% CI)**	***P***
Increasing age (+1 year)	0.98 (0.98–0.98)	<0.0001
Male	1.06 (0.96–1.21)	0.0223
Hypertension history	1.03 (0.90–1.17)	0.72
Diabetes Mellitus	1.04 (0.91–1.19)	0.53
Myocardial Infarction	0.83 (0.70–0.98)	0.027
Atrial fibrillation	0.47 (0.40–0.55)	<0.0001
Previous stroke	1.79 (1.58–2.02)	<0.0001
Statin	0.73 (0.63–0.84)	<0.0001
Antihypertensive EC-stabilizer	0.93 (0.83–1.06)	0.27

*EC, Endothelial cell; HT, hypertensive*.

**Table 5:** Multivariable model (logistic regression) comparing all patients describing odds ratio of lacunar stroke where endothelial cell stabilizers include both antihypertensive and statin medications.

	**Odds ratio (95% CI)**	***P***
Increasing age (+1 year)	0.98 (0.97–0.98)	<0.0001
Male	1.07 (0.96–1.20)	023
Hypertension history	1.04 (0.91–1.19)	0.55
Diabetes Mellitus	1.05 (0.91–1.20)	0.52
Myocardial Infarct	0.81 (0.69–0.96)	0.014
Atrial fibrillation	0.48 (0.41–0.56)	<0.0001
Previous stroke	1.80 (1.59–2.03)	<0.0001
EC stabilizer—both statin and anti-HT combined	0.83 (0.73–0.93)	0.001

*EC, Endothelial cell; HT, hypertensive*.

To assess whether there was also an association between exposure to EC-stabilizing medication and clinical outcome we performed a *post-hoc* exploratory analysis. For this, we used data for 90 day mortality post stroke. There was no change in mortality associated with taking an anti-hypertensive EC-stabilizing medication but exposure to statins was associated with a significant increased odds of being alive at 90 days (O.R. 1.49, 95% C.I.: 1.26–1.76). When we ran the analysis again, this time including statin exposure in the EC stabilizing group a reduction in 90 day mortality was seen for those taking EC stabilizing medications (O.R. 1.15, 95% C.I.: 1.00–1.32; Table 6).

**Table 6:** Multivariable model (logistic regression) describing 90 day post-stroke mortality.

	**Odds ratio (95% CI)**	***P***
Increasing age (+1 year)	1.05 (1.04–1.05)	<0.0001
Female	0.87 (0.77–0.98)	<0.0001
Hypertension history	0.91 (0.78–1.05)	0.19
Diabetes mellitus	0.82 (0.71–0.94)	0.006
Myocardial Infarct	0.68 (0.58–0.81)	<0.0001
Atrial fibrillation	0.69 (0.61–0.79)	<0.0001
Previous stroke	0.86 (0.76–0.99)	0.029
EC anti–HT stabilizer	1.15 (1.00–1.32)	0.047
Lacunar stroke	4.10 (3.28–5.21)	<0.0001

*Data presented are odds of being alive at 90 days follow-up*.

*EC anti-HT stabilizer - endothelial cell-stabilizing antihypertensive medication and/or statin medication*.

## Discussion

Due to our previous findings of EC-stabilizing medication reversing SVD pathology in a rat model (9), we took a novel approach of using pre-existing stroke trial data to test for an association between exposure to EC-stabilizing drugs and lacunar stroke in humans, allowing us to search a large amount of data. We demonstrated an association between use of these drugs and reduction in lacunar stroke and therefore by extrapolation a potential protective effect of these medications on SVD. This effect appears to be independent of reducing hypertension and lends further support to a role for EC dysfunction in the pathogenesis of SVD.

When designing the study, we recognized that a retrospective secondary analysis of clinical trial datasets had potential for bias and confounding. In particular, medications with EC-stabilizing effects are commonly prescribed in large vessel and cardiac disease. Our initial unadjusted analysis confirmed that cardiovascular risk factors were associated with lacunar stroke and had to be included in any model exploring the relationship between EC-stabilization and SVD. As SVD is associated with hypertension and as antihypertensives were our primary medication exposure of interest, we accounted for antihypertensive confounding using two methods; we included history of hypertension in our models and also restricted analyses to a subgroup of the population, removing those who had no exposure to any antihypertensive.

These initial analyses, considering EC-stabilizing medication exposure based on antihypertensive medication history only, gave unexpected and seemingly conflicting results. We had pre-specified statin use as a secondary analysis of interest, but the high proportion of patients in the dataset taking statins suggested that statins had to be accounted for in all our analyses. The potential beneficial effect of statins was confirmed in our revised analyses. The analyses including those taking any EC-stabilizing medication (statin and/or antihypertensive) is arguably the most robust and in this model EC-stabilizing exposure was associated with less lacunar strokes. This appeared independent of a reduction in blood pressure as there was a stronger reduction in the odds of a SVD-type stroke than in the group taking EC-stabilizing antihypertensives alone. The *post-hoc* analyses describing outcomes following stroke are aligned with our other findings and further support that statins have important effects and should be included in any study looking to describe EC-stabilizing medications. However, as a note of caution, we do not have data on cause of death as an outcome and so were unable to separate neurological from non-neurological mortality.

Our analyses demonstrate that large trial resources can be used for hypothesis testing and development and may be a potential platform for selecting agents that could be repurposed for SVD indications specifically. Our analyses also highlight important methodological considerations for future studies of EC-stabilizing medications, most notably the need to adjust analyses for other vascular risk factors and the limitations of considering a single drug group in isolation. Polypharmacy was the norm in this dataset, a feature also seen in real world clinical practice. While we strived for scientific purity in our initial analyses restricted to antihypertensives only, it was only when we also included statins that the results became consistent.

Secondary analysis of existing data is an approach with many caveats to its interpretation. However, using data in this way offers cost and time efficiency, allowing hypothesis exploration in a large cohort of well-phenotyped patients. Many of our results are in keeping with our current understanding of stroke epidemiology and suggest face validity of our analyses, for example the consistent strong negative association of atrial fibrillation and lacunar stroke.

We recognize the limitations of such analyses. Our VISTA data (22) contained information only on patients in stroke trials, therefore we could only assess one stroke type against another. The ideal would be a primary intervention study using prospective follow-up of a healthy cohort treated or untreated with EC-stabilizing medications with incidence of SVD as an outcome. Such primary outcome data may be obtainable in time from the UK Biobank (http://www.ukbiobank.ac.uk/) or other large prospective population studies when numbers allow. Furthermore, we recognize that there are many approaches to assessing for associations in observational data. Other approaches would include matching or more sophisticated propensity scoring, but ultimately there is no perfect analysis and residual bias is always a possibility when assessing observational data to make causal inferences. We also accept that we may be underestimating the incidence of SVD by using clinical lacunar stroke as a proxy, however, it is at least a robust outcome measure. SVD also causes the symptoms of cognitive decline and dementia (24), which we were unable to identify in this dataset, but which would be interesting to include in a prospective dataset. We did not have available dose or exposure duration data for these medications, but had to pragmatically assign exposure as “yes” or “no”: a dose response would increase our confidence in this association. Association is not synonymous with causation but our results align with evidence from animal studies in models of SVD and from human post mortem brain.

Small vessel disease (SVD) and associated lacunar strokes have been under-studied and yet the suggestion that this mechanism of disease is reversible in rat models (9) and in humans (25) could have major socio-economic impact. If the results of our analyses are proven to be true then there is the potential that standard, safe, and cheap medications may be used to reduce the physical and cognitive disability of progressive cerebral SVD. This is particularly important as to date we have no proven therapies for SVD, and only limited neuroradiological proof in humans that this may be reversible (25). Based on our data and other emerging preclinical and neuroimaging studies, we now at least have logic in moving to prospective interventional studies and this should be the next step for this common disease.

## Data Availability Statement

The datasets generated for this study are available on request to the corresponding author.

## Ethics Statement

The studies involving human participants were reviewed and approved by University of Glasgow, MVLS ethics. The patients/participants provided their written informed consent to participate in this study.

## Author Contributions

CB performed the data sorting and analyses, with the help of TQ and AW. The paper was written and edited by all authors.

### Conflict of Interest

TQ has received investigator-initiated funding, travel, or educational support from Bayer; BMS/Pfizer Alliance. AW has received research funding from Roche. The remaining author declares that the research was conducted in the absence of any commercial or financial relationships that could be construed as a potential conflict of interest.
