# Supplementary material for: Association Between Endothelial Cell Stabilizing Medication and Small Vessel Disease Stroke: A Case-Control Study
Source: Front Neurol. 2019 Sep 25;10:1029. doi: 10.3389/fneur.2019.01029 (PMC6773869; doi:10.3389/fneur.2019.01029)
Supplement: Supplementary file 1 [file Data_Sheet_1.docx]

Supplementary Material

# Supplementary Data

Supplementary Table 1:

Anti-hypertensive medications found in the VISTA dataset, divided into those with EC-stabilizing properties (described anywhere in the literature), and those without (no description of these properties in the literature).

| **EC-stabilizing properties (defined as increasing NO)** | **No EC-stabilizing properties** |
| --- | --- |
| **ACEIs:**  [Captopril](https://en.wikipedia.org/wiki/Captopril)  [Enalapril](https://en.wikipedia.org/wiki/Enalapril)  [Fosinopril](https://en.wikipedia.org/wiki/Fosinopril)  [Lisinopril](https://en.wikipedia.org/wiki/Lisinopril)  [Perindopril](https://en.wikipedia.org/wiki/Perindopril)  [Quinapril](https://en.wikipedia.org/wiki/Quinapril)  [Ramipril](https://en.wikipedia.org/wiki/Ramipril)  [Trandolapril](https://en.wikipedia.org/wiki/Trandolapril)  [Benazepril](https://en.wikipedia.org/wiki/Benazepril)  Cilazapril  Delapril  Imidapril  Moexipril  Spirapril  Zofenopril | **Diuretics:**  Furosemide  Bendroflumethiazide  Bumetanide  Torsemide  Hydrochlorothiazide and Chlorothiazide  Epitizide  Indapamide (and its derivative Metipamide )  Metolazone  Amiloride  Triamterene  Pretanide  Clopamide  Methyclothiazide  Mefruside  Cyclopenthiazide |
| **Some Beta blockers** (third generation with NO release)  [Carvedilol](https://en.wikipedia.org/wiki/Carvedilol)  [Nebivolol](https://en.wikipedia.org/wiki/Nebivolol)  [Bucindolol](https://en.wikipedia.org/wiki/Bucindolol)  [Labetalol](https://en.wikipedia.org/wiki/Labetalol)  Celiprolol | **Most beta blockers:**  [Atenolol](https://en.wikipedia.org/wiki/Atenolol)  [Nadolol](https://en.wikipedia.org/wiki/Nadolol)  [Propranolol](https://en.wikipedia.org/wiki/Propranolol)  [Timolol](https://en.wikipedia.org/wiki/Timolol)  [Metoprolol](https://en.wikipedia.org/wiki/Metoprolol)  [Oxprenolol](https://en.wikipedia.org/wiki/Oxprenolol)  [Pindolol](https://en.wikipedia.org/wiki/Pindolol)  Bisoprolol |
| **ARBs:**  [Candesartan](https://en.wikipedia.org/wiki/Candesartan)  [Eprosartan](https://en.wikipedia.org/wiki/Eprosartan)  [Irbesartan](https://en.wikipedia.org/wiki/Irbesartan)  [Losartan](https://en.wikipedia.org/wiki/Losartan)  [Olmesartan](https://en.wikipedia.org/wiki/Olmesartan)  [Telmisartan](https://en.wikipedia.org/wiki/Telmisartan)  [Valsartan](https://en.wikipedia.org/wiki/Valsartan)  [Fimasartan](https://en.wikipedia.org/wiki/Fimasartan) | Acebutolol  Esmolol  Betaxolol |
| **Calcium Channel Blockers:**  [Amlodipine](https://en.wikipedia.org/wiki/Amlodipine)  [Cilnidipine](https://en.wikipedia.org/wiki/Cilnidipine)  [Felodipine](https://en.wikipedia.org/wiki/Felodipine)  [Isradipine](https://en.wikipedia.org/wiki/Isradipine)  [Lercanidipine](https://en.wikipedia.org/wiki/Lercanidipine)  [Levamlodipine](https://en.wikipedia.org/wiki/Levamlodipine)  [Nicardipine](https://en.wikipedia.org/wiki/Nicardipine)  [Nifedipine](https://en.wikipedia.org/wiki/Nifedipine)  [Nimodipine](https://en.wikipedia.org/wiki/Nimodipine)  [Nitrendipine](https://en.wikipedia.org/wiki/Nitrendipine)  [Diltiazem](https://en.wikipedia.org/wiki/Diltiazem)  [Verapamil](https://en.wikipedia.org/wiki/Verapamil)  Nisoldipine  Manidipine  Lacidipine  Barnidipine  Nilvadipine  Mibefradil  Gallopamil  Bepridil |  |
| **Special diuretic:**  Chlorthalidone |  |
| Cicletanine | **Alpha-blockers:**  [Prazosin](https://en.wikipedia.org/wiki/Prazosin)  [Terazosin](https://en.wikipedia.org/wiki/Terazosin)  [Doxazosin](https://en.wikipedia.org/wiki/Doxazosin)  [Clonidine](https://en.wikipedia.org/wiki/Clonidine)  [Phentolamine](https://en.wikipedia.org/wiki/Phentolamine)  [Indoramin](https://en.wikipedia.org/wiki/Indoramin)  [Phenoxybenzamine](https://en.wikipedia.org/wiki/Phenoxybenzamine)  Urapidil  Rilmenidine |
|  | [Reserpine](https://en.wikipedia.org/wiki/Reserpine) |
|  | [Tolazoline](https://en.wikipedia.org/wiki/Tolazoline) |
|  | [Hydralazine](https://en.wikipedia.org/wiki/Hydralazine)  [Methyldopa](https://en.wikipedia.org/wiki/Methyldopa) |
|  | [Eplerenone](https://en.wikipedia.org/wiki/Eplerenone)  [Spironolactone](https://en.wikipedia.org/wiki/Spironolactone) (and metabolite  *potassium canrenoate*) |
| [Aliskiren](https://en.wikipedia.org/wiki/Aliskiren) | [Sodium Nitroprusside](https://en.wikipedia.org/wiki/Sodium_nitroprusside) |
| [Bosentan](https://en.wikipedia.org/wiki/Bosentan) | Ethacrynic Acid |
|  | [Guanabenz](https://en.wikipedia.org/wiki/Guanabenz)  [Guanfacine](https://en.wikipedia.org/wiki/Guanfacine)  [Guanethidine](https://en.wikipedia.org/wiki/Guanethidine) |
|  | [Moxonidine](https://en.wikipedia.org/wiki/Moxonidine)  [Mecamylamine](https://en.wikipedia.org/wiki/Mecamylamine) |

**Supplementary Table 2:**

Statin medications taken by patients in the VISTA dataset.

| Statins in dataset: |
| --- |
| Atorvastatin  Fluvastatin  Lovastatin  Pravastatin  Rosuvastatin  Simvastatin  Pitavastatin  Cerivastatin  Mevastatin  [Pitavastatin](http://www.statinanswers.com/pitavastatin.htm) |

**Supplementary Table 3.** Univariable analysis comparing all patients exposed or not to endothelial cell-stabilizing antihypertensive medication limited to patients taking antihypertensive medication

|  | Exposed to EC-stabilizing anti-HT drug  N=6671 | Exposed to non-EC stabilizing anti-HT drugs  N=1354 | P |
| --- | --- | --- | --- |
| Female | 3024 | 633 | 0.33 |
| Hypertension history | 4840 | 791 | <0.0001 |
| Diabetes Mellitus | 1456 | 204 | <0.0001 |
| Myocardial Infarct | 833 | 172 | 0.76 |
| Atrial fibrillation | 1649 | 372 | 0.23 |
| Previous stroke | 1400 | 275 | 0.24 |
|  | | | |
| Lacunar stroke | 1515 (23%) | 179 (15%) | <0.0001 |
| Non-lacunar stroke | 5156 (77%) | 1175 (85%) |  |

EC – Endothelial cell, HT-hypertensive

*The final cell reports significance testing for comparative analysis comparing proportion of lacunar stroke in those exposed and non-exposed to EC stabilizing antihypertensive medication.*
